# Autoantibody Response to ZRF1 and KRR1 SEREX Antigens in Patients with Breast Tumors of Different Histological Types and Grades

**DOI:** 10.1155/2016/5128720

**Published:** 2016-10-25

**Authors:** Lada Dyachenko, Kristina Havrysh, Anita Lytovchenko, Irina Dosenko, Stepan Antoniuk, Valeriy Filonenko, Ramziya Kiyamova

**Affiliations:** ^1^Department of Cell Signaling, Institute of Molecular Biology and Genetics NASU, Kyiv, Ukraine; ^2^Institute of Fundamental Medicine and Biology, Kazan Federal University, Kazan, Russia; ^3^Breast Cancer Department, National Cancer Institute, Kyiv, Ukraine; ^4^Department of Oncological Pathology, Dnipropetrovsk Regional Center of Pathology, Dnipropetrovsk, Ukraine

## Abstract

*Purpose*. To investigate a frequency of antibody response to SEREX-identified medullary breast carcinoma autoantigens ZRF1 and KRR1 in sera of breast cancer patients taking into account clinical and molecular characteristics of tumors for opening of new perspectives in creation of minimally invasive immunological tests for cancer diagnostics.* Methods*. Enzyme-linked immunosorbent assay and bioinformatics analysis.* Results*. Increased frequency of antibody response was found in sera of breast cancer patients to ZRF and KRR1 antigens. The antibody response to these antigens was higher in sera of patients with invasive ductal carcinoma than in sera of patients with other histological types of breast tumors. Moreover, more frequent antibody response to ZRF antigen was found in sera of patients with less aggressive tumors. The sequence analysis of ZRF1 antigen SEREX clones obtained from cDNA libraries of different tumors demonstrates that they encode different protein isoforms. *Conclusion*. Tumor-associated antigens KRR1 and ZRF1 and their cognate autoantibodies could be considered as potential molecular markers of breast cancer which need to be further investigated.

## 1. Introduction

Breast cancer is the most common female cancer in the world with high mortality rate because of high degree of malignancy and rapid proliferation. One of the main problems in breast cancer diagnostic is that traditional diagnostic methods, including mammography, ultrasound, magnetic resonance imaging, and clinical examination, are not sufficiently effective for tumor detection [1, 2]. For example, mammography has a high rate of false-positive results and is characterized by insufficient sensitivity in some groups of women [3–5]. Such methods as ultrasound and magnetic resonance imaging are used as additional tools for tumors detection [3]. Moreover, some tumors can appear in periods between annual mammography screenings being difficult for detection by widely used screening technologies. So there is a need to search for new approaches including cancer biomarkers for breast tumors detection that could provide more effective, simple, and noninvasive diagnostic tools.

Recent advances in identification and characterization of tumor-associated antigens (TAAs) indicate that their correspondent autoantibodies are perspective cancer biomarkers, which have significant advantages compared to other biomarkers, in particular, the ability to be detected long before clinical manifestation of the disease and the stability and availability in blood serum, which open up opportunities to create minimally invasive cancer diagnostic immune assays [5, 6]. According to the literature data, the detection rate of autoantibodies directed against single antigen in the sera of patients is low and ranges vary in average from 10 to 30%. Therefore, the use of panel of TAAs, rather than individual TAAs, enhances the likelihood of cancer detection. It should be mentioned that a great number of antigenic panels created for breast cancer detection consist of well-known breast cancer TAAs, in particular p53, c-myc, Her2, Muc1, and survivin [7]. Most of them are known to be overexpressed in breast carcinoma tissues and can initiate autoimmune response [7–9]. Unfortunately, most of the known antigenic panels have insufficient sensitivity and specificity for breast cancer diagnostics, and no one of them was approved for use in clinic. The first commercial panel of antigens “early CDT lung” was approved by FDA in 2012 to detect autoantibodies directed to TAAs and assess the risk of lung cancer in the group of heavy smokers. Therefore, it is important to search for the new TAAs for creation of more sensitive antigenic panels, which will be able to discriminate cancer patients and healthy individuals. Moreover, investigation of antigens immunogenicity considering clinical and molecular characteristics of tumors including stage, histological type, receptor status, and grade expands and clarifies their use as biomarkers for cancer diagnostics.

Identification and characterization of TAAs and their autoantibodies as molecular markers of human malignancies are the long-standing goal of our Cell Signaling Department. Up to date, we have identified 81 autoantigens of colon, thyroid, and breast tumors by SEREX (serological analysis of recombinant cDNA expression libraries) approach [10–17]. The preliminary phage based allogenic screening showed that 32 out of 81 SEREX-identified autoantigens had cancer-related serological profile and reacted only with sera of cancer patients [11, 15]. Among these immunoreactive autoantigens, there are 2 proteins; in particular, Zuotin-related factor 1 (ZRF1) and R motif-containing protein 1 (KRR1) (clones KY-MBC 29-88-1 and KY-MBC 18-53-1) were identified during the screening of cDNA library from medullary breast carcinoma. This study aims to confirm cancer-related serological profile of ZRF1 and KRR1 in large-scale ELISA screening of their recombinant analogues with sera of breast cancer patients of different histological types and tumor grades compared with sera of healthy individuals.

## 2. Methods

### 2.1. Blood Serum Samples of Patients

In total, 142 patients with newly diagnosed breast tumors including 22 patients with nonmalignant fibroadenoma (FA) and 120 patients with malignant tumors, namely, 87 patients who suffered from invasive ductal carcinoma (IDC), 23 who had invasive lobular carcinomas (ILC), and 10 who had medullary breast carcinomas (MBC), were recruited for this study (Table 1). 56 patients without neoplasm were involved as a control group.

Serum samples were taken by standard phlebotomy without the use of anticoagulants. Blood samples were kept at room temperature for 1 hour to form a fibrinogenic clot and then centrifuged for 15 min at 2500 ×g. Serum was pulled out, diluted with glycerol (1 : 1), divided into aliquots, and stored at −20°C.

Patient's tumors have been diagnosed using standard morphological and clinical criteria in Dnipropetrovsk City Oncology Center (Dnipropetrovs'k, Ukraine) and the National Cancer Institute, the Ministry of Health of Ukraine [18], Institute of Molecular Biology and Genetics, NASU (Kyiv, Ukraine), the Clinical Oncological Center (Dnipropetrovsk, Ukraine), and National Cancer Institute (Kyiv, Ukraine), and informed consent was obtained from all patients.

### 2.2. Cloning of TAAs cDNA in Bacteria and Purification of Correspondent Recombinant Proteins

cDNAs of antigens ZRF1 and KRR1 were amplified from original recombinant ZRF1/pBK-CMV and KRR1/pBK-CMV plasmids and inserted to pET-28b expression vector by standard cloning technique using Not1 and Sal1 exonucleases. These cDNAs which were identified during SEREX screening are not full-length but they covered the most of coding sequences (CDS) of cDNAs (Table 2). cDNAs of ZRF1 and KRR1 antigens were inserted into expression vector pET-28b in the frame with 6His-tag (Table 2) and expressed in* E. coli* Rosetta (DE3) pLysS cells. Affine purified recombinant proteins were used in ELISA screening with sera of breast tumors patients and healthy donors.

Expression of His-tag fused recombinant proteins was induced by 1 mM IPTG at 37°C for 4 h in* E. coli* Rosetta (DE3) pLysS cells transformed by correspondent recombinant plasmids. Recombinant proteins were affinity purified using Ni-NTA-agarose according to manufacturer protocol. Purity of proteins was analyzed by SDS-PAGE.

### 2.3. Enzyme-Linked Immunosorbent Assay (ELISA)

Detection of autoantibodies in sera of breast cancer patients was carried out by ELISA using 96-well plate (Sarstedt, USA). Recombinant 6His-fused proteins (0,3 *μ*g) were added to the wells in phosphate buffer (PBS), pH 7,4 (3,2 mM Na_2_HPO_4_; 0,5 mM KH_2_PO_4_; 1,3 mM KCl; 135 mM NaCl); sorption was carried out during the night at +4°C. The plates were washed with 0.1% PBS-T (phosphate buffer with 0.1% Tween), followed by blocking of binding sites with 0.1% PBS-T and 5% casein hydrolyzate (USB, USA) for 2 h at 37°C. Serum samples (at a dilution of 1 : 100 in phosphate buffer with 0.5% casein hydrolyzate) were dispensed in duplicate of 100 *μ*L per well and incubated for 90 min at 37°C. After four washings, antibodies conjugated to horseradish peroxidase (Jackson ImmunoResearch, USA) and directed against Fc-fragment of human IgG were added upon dilution of 1 : 10^4^ in phosphate buffer with 1% casein hydrolyzate and incubated for 1 h at 37°C. After washing, 100 *μ*L of chromogenic substrate ABTS (2,2′-azino-bis (3-ethylbenzothiazoline-6-sulphonic acid)) (Sigma, USA) was added per well and incubated for 30 min at 37°C. Optical density (OD) was determined at *A*_410_ using a spectrophotometer BioTek (USA).

### 2.4. Statistical Analysis

Primary data systematization and analysis were performed using Excel software (Microsoft Office, 2007). The antibody test (ELISA) was positive if optical density (OD) of serum samples exceeded the received threshold. The threshold of the test was adopted as the mean plus 2 standard deviations of the OD values of all healthy donors' samples. To determine whether the frequency of autoantibodies to each of two TAAs was significantly higher in sera of breast cancer patients compared with sera of healthy donors, data were analyzed using the chi-squared test and *t*-test. In view of great age variety in group of healthy donors and groups of breast cancer and fibroadenoma patients, the comparisons were performed between age-matched groups. For groups that include 5 or less members, statistical analysis was not executed.

### 2.5. Bioinformatics Analysis

Bioinformatic analysis of SEREX clones sequences was performed using bioinformatics tools provided by NCBI (http://www.ncbi.nlm.nih.gov/), GeneCards (http://www.genecards.org/), SEREX (http://www.ludwig-sun5.unil.ch/CancerImmunomeDB/), and CLUSTAL W (1.83) (http://embnet.vital-it.ch/software/ClustalW.html) resources.

## 3. Results

### 3.1. Cloning of cDNAs of KRR1 and ZRF1 Antigens, Expression, and Purification of Correspondent Recombinant Proteins

In this investigation, our attention was focused on two potential tumor-associated antigens, namely, Zuotin-related factor 1 (ZRF1) and R motif-containing protein 1 (KRR1), previously identified by SEREX analysis of medullary breast carcinoma cDNA library [12, 15]. These antigens had tumor-associated serological profile of autoantibodies according to the data of preliminary phage based allogeneic screening with sera of medullary breast carcinoma patients [12]. Recombinant plasmids ZRF1/pBK-CMV and KRR1/pBK-CMV were obtained by “*in vivo* excision” procedure of cDNA of phage *λ* positive clones of MBC cDNA library during previous investigation [12, 15].

### 3.2. Analysis of Antibody Response to KRR1 and ZRF1 Recombinant Proteins in the Sera of Breast Tumors Patients and Healthy Donors

Recombinant proteins were analyzed in the allogenic screening using sera of patients with different histological types and tumor grades compared with sera of healthy individuals. Since the most of breast patients were over 45 years of age while the most of the fibroadenoma patients and healthy individuals were under 45 years of age, we performed comparative analysis of serum reactivity in corresponding age-matched groups.  Table 3 presents the results of large-scale allogeneic screening of two investigated antigens. Increased frequency of autoantibody response to ZRF1 and KRR1 antigens was detected in 25.00% and 20.54% of breast cancer patients' sera, respectively (Table 3).

Analysis of antibody response frequency toward KRR1 and ZRF antigens in the sera of breast cancer patients taking into account histological type of tumors showed a statistically significant antibody response only in sera of patients with invasive ductal carcinomas. For ZRF1, significant reactivity also was shown in sera of patients with G1 and G2 tumors (Table 4).

We found no significant difference of frequency of antibody response in sera of breast cancer patients taking into account tumor's receptor and lymphoid nodes status.

As it was mentioned above, ZRF1 (also known as DNAJC2, MPP11) was described as SEREX-antigen in different malignancies [19–22]. Totally, 4 SEREX clones NY-SCLC-6, NY-BR-13, NGO-St-58 5′, and UL-CML-1 have been identified by screening of cDNA libraries from lung, breast, and stomach cancers and leukemia correspondingly (Table 5). Analysis of antibody response in sera of cancer patients toward the protein products of these clones according to literature data and our own results showed contradictory outcomes (Table 5). These data prompted us to perform analysis of available sequence of SEREX clones which encode ZRF1 (DNAJC2, MPP11) antigen.

### 3.3. Analysis of Sequences of SEREX Clones Identified from Different Tumors, Which Encode ZRF1 Antigen

Today there are three available cDNA sequences of ZRF1 SEREX clones: NGO-St-58, NY-BR-13, and KY-MBC 29-88-1. The NY-BR-13 clone was identified by Matthew Scanlan (Ludwig Institute for Cancer Research, NY, USA) by screening of breast cancer cDNA library and NGO-St-58 5′ clone was identified by Yuichi Obata (Aichi Cancer Center, Tokyo, Japan) by screening of stomach cancer cDNA library (Table 5).

**Figure 1:**
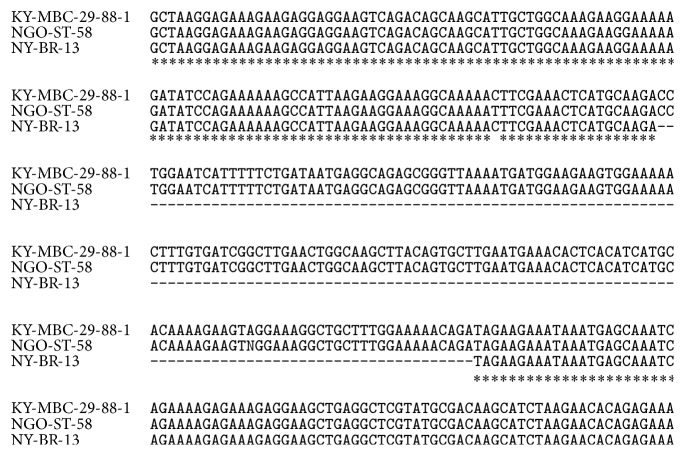
Part of multiple alignment of KY-MBC 29-88-1, NGO-St-58, and NY-BR-13 clones sequences by CLUSTAL W (1.83) analysis.

Comparative analysis of sequences of these clones revealed 159 bp length insertions in the sequences of clones NGO-St-58 and KY-MBC 29-88-1 that was absent in the sequence of NY-BR-13 clone (Figure 1). BLAST analysis of cDNA sequences of NY-BR-13, NGO-St-58, and KY-MBC 29-88-1 clones showed that sequences of clones NGO-St-58 and KY-MBC 29-88-1 correspond to the longer mRNA transcript 1 of* DNAJC2* gene (Ref. sec.* NM_014377.1*), while full-length sequence of NY-BR-13 clone (reference sequence X98260) corresponds to the shorter mRNA transcript 2 of* DNAJC2* gene (Ref. sec.* NM_001129887.1*) that differs by 159 bp (Table 6). Unfortunately, we could not analyze sequences of UL-CML-1 and NY-SCLC-6 clones because they were not found [19, 22].

Obtained data indicate that at least three SEREX clones KY-MBC 29-88-1, NGO-St-58, and NY-BR-13 correspond to different mRNA transcripts of* DNAJC2* gene, which encode in turn two different protein isoforms (Table 6).

## 4. Discussion

Tumor-associated antigens and their correspondent autoantibodies are promising molecular markers for diagnostics and therapy of human malignancy. Today more than 2000 TAAs have been identified by different approaches including SEREX, SEPRA, phage display, microarray, and other high throughput technologies [14, 23]. Previously, we have described 81 autoantigens identified from breast, colon, and thyroid cancers by SEREX approach [10, 12, 13, 15, 16]. Original modification of SEREX technique for the generation of medullary breast carcinoma cDNA expression library depleted from IgG genes has been developed [12]. As a result, MBC cDNA library suitable for serological screening was created and 41 MBC-derived autoantigens have been identified [12]. Preliminary phage based allogenic screening revealed that 18 of them had exhibited cancer-related autoantibody profile and reacted only with sera of medullary breast carcinoma patients but not with sera of healthy donors [12, 15]. Here, we studied immunogenicity of 2 out of 18 MBC antigens, namely, ZRF1 and KRR1, in sera of patients with breast tumors of different histological types and grades compared with sera of age-matched healthy donors using large-scale allogeneic screening performed by ELISA.

Zuotin-related factor 1 (ZRF1) and R motif-containing protein 1 (KRR1) have a multifunctional role in different cell processes including malignant transformation. KRR1 is a factor involved in a process of ribosomal assembly and essential for cell viability [24]. Some data demonstrate that KRR1 is highly expressed in dividing cells; moreover, its expression ceases almost completely, when cells enter the stationary phase.* In vivo* depletion of KRR1 leads to vigorous reduction of 40S ribosomal subunits due to defective 18S rRNA synthesis [24]. There is a little data about KRR1 function in tumors. It was suggested only that KRR1 is associated with metastasis in malignant fibrous histiocytoma [23]. During this study, we identified for the first time increased frequency of antibody response toward KRR1 antigen in sera of breast cancer patients and in sera of patients with invasive ductal breast carcinoma compared with sera of healthy women.

Zuotin-related factor 1 (ZRF1), that has several names including DNAJC2 (Hsp40); ZUO1; MPP11; MPHOSPH11 and is encoded by* DNAJC2* gene, is a member of the M-phase phosphoprotein (MPP) family, which acts as both a chaperone in the cytosol [25] and a chromatin regulator in the nucleus [26]. The chromosomal aberrations involving this gene are associated with primary head and neck squamous cell carcinomas (HNSCC) [27]. A tumor-specific high level of MPP11 expression in HNSCC was shown [27]. Cluster analysis showed that gene, which is EST, is moderately similar to ZFR1, displayed differential expression in lymph node positive and negative gastric tumors [28]. The mutation in the human Mpp11 J protein (ZRF1) which leads to loss of function of this protein has been described [25]. Some authors concluded that ZRF1 involved in regulation of cellular proliferation and senescence and alterations in ZRF1 may contribute to tumorigenesis [29]. Analysis performed during this study showed increased frequency of antibody response toward ZRF1 antigen in sera of patients with invasive ductal carcinomas. Greiner et al. [22] also demonstrated significant antibody response toward MPP11 in the sera of breast cancer patients but not in sera of healthy volunteers. These data contradict the data of Obata et al. [21] and Scanlan et al. [20] who both failed to show increased immunogenicity of ZRF1 antigen in sera of stomach and breast cancer patients correspondingly compared with sera of healthy women. This concordance was explained by Greiner in part by different cDNAs lengths of SEREX clones that might result in a various conformation of the gene's protein products and subsequently in a different epitope presentation to IgG antibodies [22].

We performed analysis of SEREX clones (NY-BR-13, NGO-St-58, and KY-MBC 29-88-1) sequences coding ZRF1 and revealed 159 bp lengths insert in the sequences of clones NGO-St-58 and KY-MBC 29-88-1. BLAST analysis indicated that sequences of clones NGO-St-58 and KY-MBC 29-88-1 correspond to longer transcript variant 1 of* DNAJC2* gene, while sequence of NY-BR-13 clone corresponds to shorter transcript variant 2 of* DNAJC2* gene. Thus, these data allowed us to suppose that NGO-St-58, KY-MBC 29-88-1, and NY-BR-13 SEREX clones correspond to 2 alternatively spliced mRNA isoforms of* DNAJC2* gene, that encode two different protein isoforms. This observation, in part, can explain differences in allogenic screening results of these SEREX clones performed by different researchers. Due to previous literature data and data obtained in this study, we suggest that different protein isoforms and/or mutant forms of DNAJC2 could be differently expressed in various tumors and may differ in immunogenicity. In addition, increased frequency of antibody response in sera of patients with less aggressive tumors (G1 and G2) shown during this study allowed us to suppose that autoantibodies toward ZFR1 antigen may appear at the initial steps of breast cancer malignancy and represent potential markers for early cancer diagnostics.

So, analysis of antibody response in sera of breast cancer patients with tumors of different clinical and molecular characteristics showed that KRR1 and ZRF1 antigens and autoantibodies thereto are potential breast tumor markers, which could be important for creation of new antigenic/autoantibody signatures for breast tumor detection including early breast cancer diagnostics and/or for improving already existing ones. Comparative analysis of ZRF1 SEREX clones showed that they code different protein isoforms of* DNAJC2* gene in different tumors and cell lines.

## Figures and Tables

**Table 1:** Characteristics of tumor patients and healthy donors.

Characteristics	Samples description
(1) Group of healthy donors	
Number of healthy women	56
Age at diagnosis, years (range, mean ± SD)	17–60, 40,11 ± 12,20
The number of healthy donors 45 years old or older	21
The number of healthy donors younger than 45 years	35
(2) Group of patients with benign breast tumors	
Number of fibroadenoma patients	22
Age at diagnosis, years (range, mean ± SD)	24–63, 30 ± 9,54
The number of fibroadenoma patients 45 years old or older	2
The number of fibroadenoma patients younger than 45 years	20
(3) Group of cancer patients	
Number of patients	120
Age at diagnosis, years (range, mean ± SD)	36–83, 59.55 ± 11,63
The number of cancer patients 45 years old or older	112
The number of cancer patients younger than 45 years	8
Tumor type	
Invasive ductal breast carcinoma	87
Invasive lobular breast carcinoma	23
Medullary breast cancer	10
Tumor grade (% from general)	83
	Grade 1: 4,82
	Grade 2: 37,35
	Grade 3: 57,83
ER-status (%)	52
Positive	71,1
Negative	28,9
PR-status (%)	52
Positive	30,8
Negative	69,2
HER-2/neu status (%)	52
Positive	30,8
Negative	69,2
Lymphoid nodes status (% of positive)	28,8

SD: standard deviation.

**Table 2:** Antigens included in serological screening.

Antigen	Full name	cDNA reference sequence (NCBI db)	cDNA-fragment according to correspondent NCBI reference sequence, bp	Vector	MW of recombinant protein, kDa (with 6His-tag)
ZRF1	Zuotin-related factor 1	NM 014377.1 CDS 252⋯2117	942–2117	pET-28b (6His-tag)	47,52
KRR1	R motif-containing protein 1	NM 007043.6 CDS 42⋯1187	42–1161	pET-28b (6His-tag)	46,31

**Table 3:** Frequency of antibody response toward KRR1 and ZRF1 antigens in the sera of patients of different histological types of breast tumors in view of age^*∗*^.

Antigen	The number of positive sera^*∗∗*^, *n* (%)						
	HD over 45 (*n* = 21)	HD under 45 (*n* = 35)	BC over 45 (*n* = 112)	MBC over 45 (*n* = 7)	IDC over 45 (*n* = 82)	ILC over 45 (*n* = 23)	FA under 45 (*n* = 20)
*ZRF1*	*1 (4,76)*	*1 (2.86)*	*28 (25,00)* ^†^	*1 (14,29)*	*22 (26,83)* ^†^	*5 (21,74)*	*2 (10,00)*
Mean ± SD	0,1513 ± 0,057	0,1446 ± 0,049	0,2242 ± 0,132	0,20534 ± 0,061	0,2383 ± 0,144	0,18 ± 0,084	0,1706 ± 0,094
*KRR1*	*0 (0)*	*2 (5,71)*	*23 (20,54)* ^†^	*1 (14,29)*	*21 (25,61)* ^†^	*1 (7,14)*	*0 (0)*
Mean ± SD	0,2288 ± 0,080	0,2403 ± 0,060	0,3246 + 0,080	0,2843 ± 0,072	0,3430 ± 0,077	0,2712 ± 0,068	0,1884 ± 0,054

Notes: ^*∗*^statistical analysis of frequency of antibody response was calculated between BC patients and healthy individuals cohorts over 45 years old and between FA and healthy individuals cohorts under 45 years old; ^*∗∗*^threshold: the mean plus 2 SD in the group of healthy donors; ^†^Statistically significant difference compared to healthy donors' sera (*P* < 0.05). HD: healthy donors; BC: breast cancer (IDC + ILC + MBC); MBC: medullary breast carcinoma; IDC: invasive ductal carcinoma; ILC: invasive lobular carcinoma; FA: fibroadenoma.

**Table 4:** Frequency of antibody response toward KRR1 and ZRF1 antigens in the sera of breast cancer patients of different tumor grade.

Tumor grade (*n*: the number of positive sera^*∗*^)	Antigens	
	ZRF1	KRR1
G1 + G2 (*n* = 35)	42,86 (15)^†^	20 (7)
G3 (*n* = 48)	18,75 (9)	18,75 (9)

G1: grade 1 tumor, G2: grade 2 tumor, and G3: grade 3 tumor; *n*: the number of positive sera; ^*∗*^threshold: the mean plus 2 SD in the group of healthy donors. ^†^Statistically significant difference compared to G3 tumor grade (*P* < 0.05).

**Table 5:** SEREX clones which encode ZRF1 (DNAJC2, MPP11) antigen.

Clone name	Source	Frequency of antibody response in cancer patients sera versus healthy donors sera	Publication
NY-SCLC-6	NCI-H740 and SK-LC-13 cell lines	N/A	Güre et al., PNAS, 2000; 97: 4198–4203 [19]
NY-BR-13	Breast cancer	3/10 BC versus 3/12 HD	Scanlan et al., Cancer Immun 2001: 4–21 [20]. (Ludwig-sun5.unil.ch/CancerImmunomeDB/)
NGO-St-58, 5	Stomach cancer	4/13 SC versus 3/16 HD	Obata et al. (Ludwig-sun5.unil.ch/CancerImmunomeDB/) [21]
UL-CML-1	K562 cell line from CML patient	7/19 AML, 6/16 CML, 4/10 RCC, 3/6 MM, 3/12 BC, 3/10 OC versus 0/20 HD	Greiner et al., Int. J. Cancer 2003; 106: 224–231 [22].
KY-MBC 29-88-1	Breast cancer	33/120 BC (including 1/10 MBC, 27/87 IDC, 5/23 ILC), 3/22 FA versus 2/50 HD	Present paper

BC: breast cancer patients; HD: healthy donors; SC: stomach cancer; RCC: renal cell carcinoma; MM: metastatic melanoma; AML: acute myeloid leukemia; CML: chronic myeloid leukemia; OC: ovarian carcinoma.

**Table 6:** Comparative analysis of SEREX clones which encode ZRF1 (DNAJC2, MPP11) antigen.

SEREX clones	mRNAs Ref. Seq. for DNAJC2 gene	Gene synonym	mRNA length, bp	cds	protein_ID	Protein length, aa
NGO-St-58, 5′ KY-MBC 29-88-1, 5′	*NM_014377.1* * Homo sapiens* DnaJ (Hsp40) homolog, subfamily C, member 1 (DNAJC2), transcript variant 1, mRNA	MPHOSPH11; MPP11; ZRF1; ZUO1	2212	252⋯2117	*NP_055192.1* dnaJ homolog subfamily C member 2 isoform 1 [H. sapiens]	621

NY-BR-13 X98260	*NM_001129887.1* * Homo sapiens* DnaJ (Hsp40) homolog, subfamily C, member 2 (DNAJC2), transcript variant 2, mRNA	MPHOSPH11; MPP11; ZRF1; ZUO1	2053	252⋯1958	*NP_001123359.1* dnaJ homolog subfamily C member 2 isoform 2 [H. sapiens]	568

Notes: Ref. Seq.: Reference Sequence; bp: base pair; aa: amino acids; cds: coding sequence; ID: identification code.
